# Dosing down with biologic therapies: a systematic review and clinicians’ perspective

**DOI:** 10.1093/rheumatology/kew464

**Published:** 2017-02-16

**Authors:** Christopher J Edwards, Bruno Fautrel, Hendrik Schulze-Koops, Tom W J Huizinga, Klaus Kruger

**Affiliations:** 1 Musculoskeletal Research Unit, NIHR Wellcome Trust Clinical Research Facility, University Hospital Southampton NHS Foundation Trust, Southampton, UK; 2 Pierre et Marie Curie University – Paris 6, Sorbonne Universités, Pierre Louis Institute of Epidemiology and Public Health, Paris, GRC-08 (EEMOIS); 3 APHP, Rheumatology Department, Pitié Salpêtrière Hospital, Paris, F-75013, France; 4 Division of Rheumatology and Clinical Immunology, Department of Medicine IV, Ludwig-Maximilians-Universität, Munich, Germany; 5 Department of Rheumatology, Leiden University Medical Center, Leiden, C1-41, The Netherlands; 6 Rheumatologisches Schwerpunktzentrum, Munich, Germany

**Keywords:** systematic review, biologic therapy, dose tapering, dosing down, treatment withdrawal, dose spacing, rheumatoid arthritis, axial spondyloarthritis, psoriatic arthritis

## Abstract

The effectiveness of biologic therapies now means that remission or low disease activity are realistic targets for treatment. However, after achieving remission/low disease activity, the next steps remain unclear. The aim of this publication was to conduct a broad systematic literature review to evaluate dosing down of biologics. After screening papers and abstracts for relevance and application of inclusion/exclusion criteria, a structured extraction process was used to collect information on the included studies. Fifty-two papers were included in the analysis across rheumatic disease. In patients who discontinue therapy, remission is not typically sustained, with reported rates of relapse and flare across early RA (48–54%), established RA (2–84%), axial spondyloarthritis (11–53%) and PsA (44.9%). In many cases, an acceptable disease activity can be regained upon retreatment. More research is needed to understand the long-term impacts of these strategies on efficacy, safety and cost.

Rheumatology key messages

- Even in established RA, careful and controlled dose reduction appears possible for some individuals.
- Examination of disease state and personal characteristics is needed for determining suitability for dosing down.
- Most patients respond well on reintroduction of treatment following flare after dose reduction.

## Introduction

Biologic treatment for RA, PsA and axial spondyloarthritis (axial SpA) is now commonplace. International guidelines recommend biologics in patients whose RA or PsA is not adequately controlled by traditional DMARDs [1, 2], or whose axial SpA is not controlled by NSAIDs [3].

The effectiveness of biologic therapies now means that remission or low disease activity (LDA) are realistic targets for treatment for RA [4], PsA and AS [5]. Despite intense debate over definitions, remission rates in RA have doubled in the decade 2000–10 [6]. However, it is unclear whether the dose should be maintained, titrated down or withdrawn. In addition, if dose titration or withdrawal is carried out, how the patient should be monitored for ‘flare’ is debated [7–10].

Most data have been provided by randomized clinical trials (RCTs), although observational studies as well as data from registries are available. A Cochrane review of down-titration and discontinuation strategies of TNF blockers in RA that included seven clinical trials of etanercept and adalimumab concluded that dose reduction of etanercept 50 mg weekly to 25 mg weekly, after at least 3–12 months of LDA, seemed as effective as continuing the standard dose, and discontinuation was inferior to continuation of treatment [11].

The purpose of our review was to evaluate evidence from all published literature in order to provide clinicians with an overview of the practical uses of dose-reduction strategies. We attempted to answer seven core questions through auditing and analysing the literature, as well as to create a research agenda for future projects and studies.

## Literature search

### Search strategy

We searched PubMed (January 2000 to October 2014), Embase (January 2000 to October 2014), Cochrane Library (January 2000 to October 2014), ACR abstracts (2013–14), EULAR abstracts (2013–14) and International Congress on Spondyloarthropathies abstracts (2014). Due to the nature of the ACR and International Congress on Spondyloarthropathies databases, hand-searching for relevant abstracts was conducted. Searches of the EULAR database were carried out with [all words] in the search tool. Table 1 gives a list of the 26 primary search terms and 21 secondary search terms used. Searches were performed using a combination of a single primary term in conjunction with each secondary term to form combinations of [primary term AND secondary term].

**Table 1:** Terms used in literature search

Primary search terms	Secondary search terms
RA	Dose titration
Axial spondyloarthritis	Dose reduction
AS	Dose de-escalation
Non-radiographic axial spondyloarthritis	Dose tapering
PsA	Spacing
Biologics	Cessation
TNF	Stopping
TNF	Interval widening
Anti-TNF	Dosing down
Anti-TNF	Treatment holiday
Adalimumab	Dose interval increase
Humira	Drug withdrawal
Etanercept	Variable dosing
Enbrel	Flexible dosing
Infliximab	Dose adjustment
Remicade	Disease flare
Abatacept	Discontinuation
Orencia	Stepwise decrease
Certolizumab	Remission
Certolizumab pegol	Optimization
Cimzia	On-demand treatment
Golimumab	
Simponi	
Tocilizumab	
Actemra	
RoActemra	
Rituximab	
Rituxan	

We included studies published in English, with primary data on adults with RA, PsA or axial SpA. An additional inclusion criterion was that the study must relate to dose tapering, with this term, or a variation of it, contained within the title or abstract. Exclusion criteria included meeting abstracts without sufficient publicly available details, studies with fewer than five patient cases and studies investigating side-effect profiles.

Screening was performed by one assessor and subsequently refined by an additional assessor. Related citations to relevant topics were also searched. Hits were manually de-duplicated across databases. To be included in the final analysis, articles had to report primary data from studies conducted in adults with RA, PsA or axial SpA; be related to a therapy listed as a primary search term in Table 1; and the issue of biologic tapering had to be mentioned in the title or abstract.

The authors discussed findings of the initial literature search and observed that some key studies were not found as a result of search terms or human error of the hand-searching of databases. In addition, key studies recently published were discussed and included although they fell outside the period of the initial search. Reasons for inclusion included further real-world evidence and biologics that were not identified in the original search, as well as additional studies in those inflammatory diseases that had few studies in the initial reference list.

### Data extraction

Two authors independently extracted data from each study using a data extraction form adapted from the Cochrane Systematic Review Extraction Form template. Differences were resolved by discussion and consensus. For the purposes of this systematic review, the definitions in Table 2 were used.

**Table 2:** Definitions used in this sysyematic review

Discontinuation	Complete withdrawal of the biologic
Tapering by dose reduction	Maintaining the same frequency of dose, but reducing the quantity of the drug per administration
Tapering by injection/ infusion frequency reduction	Maintaining the same quantity of drug per administration, but increasing the time in between injections/infusions
Progressive stepwise	Initially tapering by dose reduction or tapering by injection/infusion frequency reduction, and then further tapering again by dose reduction or frequency reduction (i.e. initially 50 mg/7 days then 25 mg/7 days then 25 mg/14 days)
Disease activity– driven tapering	The decision is made whether or not to dose-down based on the patient’s disease activity
Flare	Considered in the paper as synonymous with relapse or loss of remission/LDA or failure of the tapering strategy

### Risk of bias assessment

RCTs were assessed for methodological quality using the Cochrane Risk of Bias tool [12]. The checklist of Downs and Black was used to assess the risk of bias in observational studies [13]. The risk of bias assessment was conducted by one reviewer. A second reviewer checked the results against the source document and any discrepancies were resolved by discussion and consensus.

### Data analysis

We assembled a group of five rheumatologists with an interest of dose optimization to create core questions based on what clinical rheumatologists might commonly ask when considering dosing down a biologic. Having developed seven key questions during face-to-face meetings, we used these to direct a systematic literature review to describe the evidence behind these questions (Table 3).

**Table 3:** Seven core questions to be answered

Question No	Question
1	Does tapering of biologics occur, and what are the various strategies adopted?
2	Which disease and patient characteristics are helpful in deciding on a dose-down strategy?
3	Which therapies can be dosed down, and how should this occur?
4	How should flare be defined, and what is the risk of relapse?
5	How should patients be monitored while on tapered doses of biologics?
6	How should patients be managed long term in terms of retreatment and response?
7	What are patients’ perspectives regarding tapering of biologics and its various aspects?

**Fig. 1:**
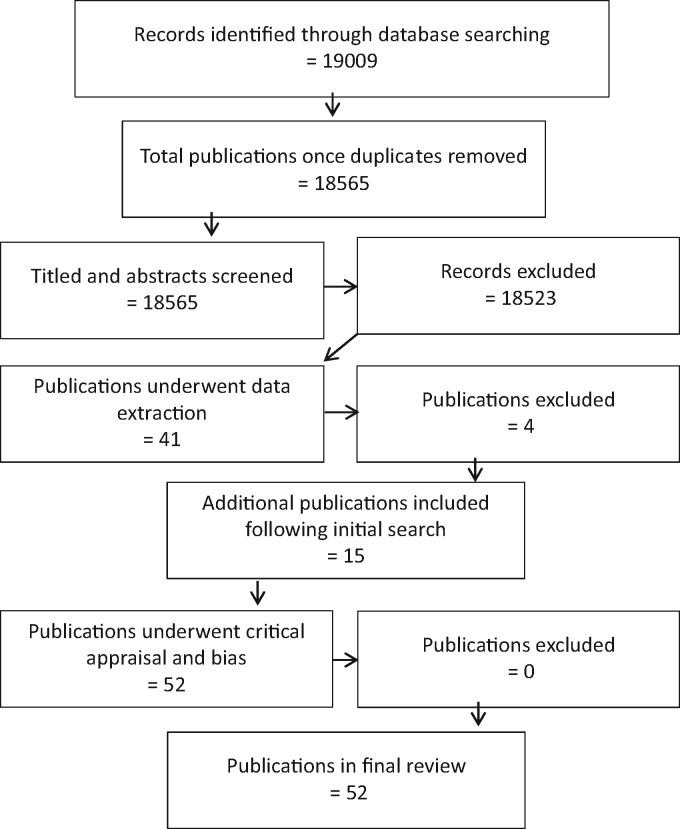
Selection of studies flowchart

A total of 52 studies underwent data extraction (Fig. 1). Among the publications we included six reporting on the BeSt Study [14–19], four on STRASS [20–23] and two each on HONOR [24, 25], van der Ven *et al.* [26, 27] and the Southampton group [28, 29]. Results obtained from the analysis were then used to help answer a number of questions (supplementary Table S1, available at *Rheumatology* Online)—each of the questions has been answered within the context of the disease type in which dosing down was studied: early RA; established RA; axial SpA; and PsA. One study had a cross-indication population, which has been taken into consideration.

## Early RA and established RA

### Does tapering of biologics occur, and what are the various strategies adopted?

#### Early RA

In the case of early RA, we found only RCTs that had investigated dosing down (50%; decrease in dose; one RCT) and discontinuation (three RCTs) [14, 30–32]. No RCT had yet investigated the strategy of injection spacing. In the case of the PRIZE study, patients who initially dosed down were given the option of continuing within the trial, then withdrawing treatment completely in a step-down process (results not discussed here) [31]. As all the studies in early RA were RCTs, the protocols adhered to were fixed with specific time points for dosing down and withdrawal [32].

#### Established RA

For established RA, nine RCTs were identified, one non-randomized trial, two placebo-controlled pilot studies, five prospective studies, nine observational studies, three reports from registries and two retrospective reports (supplementary Table S1, available at *Rheumatology* Online). A large number of different strategies have been adopted that fall within three overarching strategies: discontinuation of therapy; dose reduction—dosing down through decreasing the dose, in the majority of cases this is by 50%, but in some cases 25 or 33%; and injection frequency reduction—dosing down through increasing the spacing between individual doses, which leads to a typical decrease in dose of 50%.

In some studies a step-wise disease activity-driven protocol was adopted whereby, should the patient continue to be in remission, then an additional reduction in the dose or an increase in intervals between injections was carried out [21]. In other cases, the patients were asked to continue to increase the interval between doses until their disease flared or they could be considered for complete discontinuation of the biologic [33].

### Which disease and patient characteristics are helpful in deciding on a dose-down strategy?

#### Early RA

These RCTs had a number of criteria that had to be met in order to allow the patient to dose down their biologic treatment, but most focused around the DAS28 score. In the case of the OPTIMA and PRIZE studies, both required patients to have a DAS28 </⩽ 3.2 [31, 32]. PRIZE then went on to specify that the patients should have a DAS28 < 2.6 in order for them to withdraw treatment, regardless of the three arms they had been in during the second period of treatment (25 mg etanercept + MTX; MTX; no treatment) [31]. Minimal duration of remission or LDA was requested in only one study, the BeSt study: DAS44 ⩽2.4 for a minimum of 4 months [14].

#### Established RA

The majority of RCTs used LDA (DAS28 < 3.2) or clinical remission (DAS28 ⩽ 2.6) to define entry to dose reduction. Some studies required other DASs or multiple criteria to be met prior to dose reduction. These included: absence of synovitis on power Doppler US, absence of radiographic progression on X-ray, low or no swollen or tender joint count (SJC/TJC) compared with baseline, and no CS use [21, 26, 28].

Large variation in rates of flare/failure to prolong remission or LDA following dose-down, was observed across longstanding and early disease. Almost all studies monitored the DAS scores, with a large number taking SJC/TJC measurements and HAQ.

### Which therapies can be dosed down and how does this occur?

#### Early RA

The four RCTs investigated the dosing down of infliximab (one RCT), adalimumab (two RCTs) and etanercept (one RCT). As discussed above, the most common approach to dosing down was discontinuation in the case of all three of these biologics, with etanercept the only biologic to have been investigated in the case of a dose decrease of 50% with the same treatment interval.

#### Established RA

Across all the studies investigating dosing down approaches in the established RA population, a large spectrum of the biologics available for RA treatment have been investigated: etanercept, infliximab, adalimumab, certolizumab pegol, golimumab, tocilizumab, rituximab and abatacept. In one registry study with rituximab, the initial dose of rituximab was not clear [34]. In the RETRO trial [35], both conventional synthetic DMARD (csDMARD) and biologic DMARD (bDMARD) were tapered together. The EULAR guidelines recommend to start with tapering steroids, then bDMARD, then csDMARD [1].

### How should flare be defined, and what is the risk of relapse?

#### Early RA

In the four trials, relapse was defined as loss of remission or LDA. In the case of adalimumab, discontinuation led to 54% of patients no longer being in remission (defined in the OPTIMA study as DAS28-CRP <3.2) at week 78 [32]. In the case of etanercept, a decrease of 50% in dose led to 37% of patients no longer having a DAS28 <2.6, compared with 60 and 77% of patients who had either etanercept, or etanercept and MTX discontinued [31], respectively. In the BeSt study with infliximab, 48.1% of patients flared according to the study definition of DAS28 >2.4, with a median time of 17 months [14].

#### Established RA

Flares after stopping therapy for RA ranged between 2 and 84%. Definitions of flare included the rheumatologist’s personal discretion and measurements of disease activity passing the threshold for which the patient could no longer be defined as in remission or LDA; however, the use of US has been used as an assessment of flare.

RCTs assessing biologic tapering in patients with RA, using either a discontinuation, dose reduction or increasing injection intervals strategy, have shown varying success rates and varying relapse rates. Similar discontinuation relapse rates have been seen with infliximab and adalimumab. Likewise, the HONOR RCT [24] showed that 52% of the 52 patients who discontinued their adalimumab treatment did not sustain remission (DAS28 <2.6) at 1 year. Flares were reported in as many as 84% of cases within a year of discontinuation [36].

Increasing the interval between biologic injections has been studied by van Herwaarden *et al.*, who increased the interval of etanercept and adalimumab injections every 3 months until flare or discontinuation of drug. An investigation as to whether serum drug levels could predict the success of dose reduction or discontinuation regime found that at 18 months, dose reduction was no longer possible in 36% of patients receiving etanercept and 50% of patients receiving adalimumab.

It appears that some disease characteristics may reduce the success of the dosing-down regimen. These include a higher level of initial disease activity and presence of RF or ACPA [21, 28, 35]. DAS below 2.1 or 2.2 was shown as a predictor of success in the RRR and HONOR studies [24, 37].

RA registry data for over 700 patients showed that 72.4% of patients were no longer feeling the clinical benefit of the initial therapy after the drug had been discontinued for 36 months [38].

### How should patients be monitored while on tapered doses of biologics?

#### Early RA and established RA

A number of disease activity measures, including DAS28, along with scores of functional activity using HAQ, have been assessed. The frequency of study assessment varied from monthly to 6-monthly, and studies lasted up to 12 or 18 months [14, 31, 32].

### How should patients be managed long term in terms of retreatment and response?

#### Early RA

In the case of the BeSt study, as more long-term data are available than are available for other studies, once infliximab was re-introduced, 100% of patients achieved a DAS44 ⩽2.4. The BeSt trial utilized varying strategies, and patients had to go through several different treatment regimens to reach biologic treatment before then reaching a threshold such that infliximab could be withdrawn [14].

#### Established RA

Re-introduction of the biologic after flare resulted in many patients regaining LDA or remission. In the study of Takeuchi *et al.* [39], patients were observed to have a DAS28-CRP decrease of 1.3 within 12 weeks of the reintroduction of therapy. This speed of response was also reflected in the observational study by Brocq *et al.* [40], in which 100% of patients eventually achieved remission (defined as DAS28 <2.6), 86.7% of whom achieved remission within 2 months. Furthermore, in the HONOR study, 90% of patients achieved a LDA (defined as DAS28-ESR <3.2) within 6 months and 100% within 9 months [24].

This response rate following reintroduction of biologic therapy was not observed for all studies. In the case of the STRASS study, 40.8, 38.8 and 8.2% of patients achieved remission (DAS28 ⩽ 2.6), LDA (not defined) and moderate disease activity (not defined), respectively [21]. In the study of Marks *et al.* [28], where US remission was also one of the criteria for dosing down, 19, 19 and 47% of patients achieved DAS and US remission (DAS28 <2.6 and PDUS <1), DAS remission (DAS28 < 2.6) and LDA (DAS28 <3.2 and PDUS ⩽1), respectively [28]. Overall, following reintroduction of biologics, 19–100% of patients went on to regain remission [28, 40].

### What are patients’ perspectives regarding tapering of biologics and its various aspects?

#### Early RA and established RA

No studies have been found in this search regarding patient preference. It is the feeling of the authors that in everyday practice, tapering is often an important patient preference.

## Axial SpA

### Does tapering of biologics occur, and what are the various strategies adopted?

Tapering of biologics in patients with axial SpA has been investigated in three prospective and one observational study. In the three prospective studies, the patients were listed as having AS, whereas in the observational study, the patients were listed as having axial SpA [41–44]. Across studies, various strategies were adopted, including reducing the dose by 50% or increasing the interval. In one study by Arends *et al.* [43], if the patients maintained LDA, a progressive step-down approach was adhered to. Although exact details were not provided in the paper by Zavada *et al.* [44], the authors discuss an average dose decrease of 50% being achieved by either spacing or a reduction in the dose, reflecting an average.

### Which disease and patient characteristics are helpful in deciding on a dose-down strategy?

In two studies, the patients were required to have a BASDAI <4 for over 6 months of treatment on the biologic, reflecting an approach that requires sustained LDA before the physician will dose down [43, 44]. In other studies, BASDAI ⩽ or <2 were the requirements [41, 42]. Additional specifications were an absence of arthritis or enthesitis as well as a normal CRP level. As per the recommendations made by EULAR and ASAS, patients were initially treated with NSAIDs; this continued throughout treatment in some cases, and in other cases withdrawal of NSAIDs from the whole treatment paradigm was a necessity for dosing down.

### Which therapies can be dosed down and how does this occur?

Across all four studies in the axial SpA population, etanercept was used in all four patient populations. In three of the four studies, a portion of the investigated population received adalimumab and infliximab. In the case of etanercept, the most common manner in which this biologic was dosed down was by a dose reduction of 50% to 25 mg every week; however, other methods were investigated, including increase in spacing [41–43]. For adalimumab, spacing leading to a reduction by 50% was utilized [42]. The Arends *et al.* [43] paper, which reported on a step-wise approach, involved dose reduction by increase in spacing across etanercept, infliximab and adalimumab.

### How should flare be defined and what is the risk of relapse?

In the Arends *et al.* [43] study with the step-wise disease activity-driven approach, 26, 38, 43 and 47% of the patients no longer remained on the dose reduction method implemented as per the protocols at 6, 12, 18 and 24 months, respectively. In the other studies, the number of patients defined to have failed (varying definitions, mostly surrounding the BASDAI score that was required for the patient to be considered for dosing down) varied from 5–15% at 3 months to 16.5–29% at 6 months and 11–53% at 12 months [41, 42, 44].

### How should patients be monitored while on tapered doses of biologics?

No recommendations were reported on the specific manner in which the patients should be monitored while dosing down across all four papers. However, in most cases the patients’ BASDAI, BASFI and HAQ were followed regularly across the study duration [41, 43, 44].

### How should patients be managed long term in terms of retreatment and response?

In the case of Arends *et al.* [43], of the patients who returned to an increased dose, 88% regained a BASDAI 

<4 after 6–12 months of retreatment.

### What are patients’ perspectives regarding tapering of biologics and its various aspects?

No information is available to address this question.

## PsA

### Does tapering of biologics occur, and what are the various strategies adopted?

In the case of PsA, data are very limited regarding the dosing down of biologics. The only study found was a 2014 ACR abstract from a registry. This informs us that tapering of biologics does occur in the real-world clinical setting for PsA patients. In this case, the patients had their TNF inhibitors discontinued. In the cross-indication study, which included PsA patients, specific details were not clear for this population [45].

### Which disease and patient characteristics are helpful in deciding on a dose-down strategy?

The physicians were guided by a Clinical Disease Activity Index (CDAI) ⩽10 and a skin psoriasis physician global assessment ⩽20/100 [45].

### Which therapies can be dosed down, and how does this occur?

The abstract does not name specific biologics, but rather investigates the role of discontinuation in the TNF inhibitor class as a whole [45].

### How should flare be defined, and what is the risk of relapse?

In the registry, patients were defined as having lost clinical benefit of the initial treatment if they encountered any of the following after therapy was discontinued: CDAI >10; skin psoriasis physician global assessment >20; increase in concomitant DMARD or prednisone; starting or restarting DMARD, prednisone or biologic therapy. It was found that 44.9% of patients were regarded as having lost the benefit within a median time of 29.2 months [45].

### How should patients be monitored while on tapered doses of biologics?

No information was reported on the monitoring of these patients, with no details provided on the metrics taken or when they were taken [45].

### How should patients be managed long term in terms of retreatment and response?

No information was provided on the effect of retreatment following flare or loss of clinical benefit.

### What are patients’ perspectives regarding tapering of biologics and its various aspects?

As above, no information was available on this.

## Conclusions

Dose reduction and tapering are taking place in a number of settings, and various strategies and approaches are being adopted. There is variability in the disease and patient characteristics being used in the decision-making process, and no clear monitoring approach is in place. It seems clear that withdrawal of biologic therapy in established disease results in failure. Several guidelines and recommendations suggest cautious tapering in selected patients [1, 46], but this is not reflected in the various product licences for biologics.

It is important to understand the risks and benefits of withdrawal and dose-down strategies for biologic therapies, and the potential impacts of these approaches for both patients and health-care systems in terms of efficacy, safety and cost over both the short- and long-term. The EULAR guidelines recommend to start tapering steroids, then bDMARD, then csDMARD [1].

The results of our systematic literature review and analysis suggest that the complete withdrawal of biologic therapy in patients with established disease does not result in sustained LDA or remission, and the majority of patients will experience a flare of their disease. Flares were reported in as many as 84% of cases within a year of discontinuation [36]. However, while discontinuation of biologic therapy may not be appropriate in established disease, there may be a basis for careful and controlled dose-down or reduction in some patients because patients responded well on reintroduction of treatment. Based on these findings, it could be proposed that we define two treatment phases: a first full-dose remission-induction phase; then a remission-maintenance phase with reduced dosage or frequency, such as has been proposed in other CTDs.

In addition to standard or commonly accepted criteria for LDA or remission, we have identified several other markers that are being used to identify candidates for dose-down, such as a history of stable dosing of biologic, or a patient having no need of CSs for a defined period. Reduction in or absence of SJC, TJC or synovitis, as well as a CDAI ⩽10 and the absence of radiographic progression on X-ray have also been used as measures of eligibility. In a time of stratified medicine, RCTs often use a blanket approach to patient assessment and classification; however, some studies have allowed the evaluation of more disease activity–driven responses that are reflective of clinical practice [21, 28].

There are limitations to our analysis. The studies included are not all RCTs, and as such have been conducted with varying designs and data collection strategies, which can make drawing meaningful comparisons difficult. However, the inclusion of non-RCT data was intentional in order to capture all ideas in this rapidly changing field. As with any systematic literature review, the results will quickly be out of date with the emergence of new publications on the topic, but we hope that this offers a robust analysis of our current position and expert clinical thinking on the issues at hand.

For the future, the rise of personalized medicine calls for a bespoke approach in each patient, and the current review and analysis support the careful examination of each patient’s individual disease state and personal characteristics in order to ascertain their suitability for dose-down approaches. It is important to assess the patient’s own views and to take these into account when tailoring treatment strategies.

There are several gaps that warrant additional research in this area. Paramount is the need for assessing whether failed attempts to taper a drug cause any long-term damage in terms of immunogenicity, higher disease activity, structural damage or radiographic progression, or whether increased CRP exposure leads to a higher incidence of cardiac diseases in these patients. Of these, immunogenicity requires particular consideration, since anti-drug antibodies to TNF inhibitors are often increased in the presence of lower doses of drug, and patients with low trough levels may not represent good candidates for dose-down. Therapeutic drug monitoring is also increasingly important, and to date no study has included this in the decision criteria for dose reduction or withdrawal.

## Supplementary Material

Supplementary Table S1
