# Dosing down with biologic therapies: a systematic review and clinicians’ perspective

**DOI:** 10.1093/rheumatology/kex503

**Published:** 2017-12-12

**Authors:** Christopher J Edwards, Bruno Fautrel, Hendrik Schulze-Koops, Tom W J Huizinga, Klaus Kruger

**Affiliations:** 1 Musculoskeletal Research Unit, NIHR Wellcome Trust Clinical Research Facility, University Hospital Southampton NHS Foundation Trust, Southampton, UK; 2 Pierre et Marie Curie University Paris 6, Sorbonne Universités, Pierre Louis Institute of Epidemiology and Public Health, Paris, GRC-08 (EEMOIS); 3 APHP, Rheumatology Department, Pitié Salpêtrière Hospital, Paris, F-75013, France; 4 Division of Rheumatology and Clinical Immunology, Department of Medicine IV, Ludwig-Maximilians- Universität, Munich, Germany; 5 Department of Rheumatology, Leiden University Medical Center, Leiden, C1-41, The Netherlands; 6 Rheumatologisches Schwerpunktzentrum, Munich, Germany

Rheumatology 2017;56:1847–1856. doi:10.1093/rheumatology/kew464

 The author affiliations in this paper have been corrected online. The correct affiliation numbers are also given above.

