# Supplementary material for: Dosing down with biologic therapies: a systematic review and clinicians’ perspective
Source: Rheumatology (Oxford). 2017 Feb 16;56(11):1847–56. doi: 10.1093/rheumatology/kew464 (PMC5850865; doi:10.1093/rheumatology/kew464)
Supplement: Supplementary Table S1 [file rhe-16-0848-file002_kew464.docx]

**SUPPLEMENTARY DATA**

**Supplementary Table S1. Overview of the papers**

| **Author and year** | **Disease** | **Study design** | **N** | **Biologic (initial dose)** | **Entry criteria to dose down/discontinue** | **Dosing down method** | **Defintion of flare/ failure** | **% failure and time of assessment** | **Success after reintroduction** |
| --- | --- | --- | --- | --- | --- | --- | --- | --- | --- |
| ***Early RA*** | | | | | | | | | |
| Detert et al. 2013 (HITHARD) (30) | DMARD-naïve, early RA | RCT | 172 | ADA (40 mg eow) | Per protocol | Discontinuation | Difference in DAS between discontinue arm and no treatment | 0.21 (p=0.41) (48 weeks) | - |
| Smolen et al. 2013 (OPTIMA) (32) | DMARD-naïve, early RA | RCT | 1032 | ADA (40 mg eow) | DAS28-CRP <3.2 and PaGA | Discontinuation | DAS28-CRP <3.2) | 54% (week 78) | - |
| Emery et al. 2014 (PRIZE) (31) | DMARD-naïve, early RA | RCT |  | ETN (50 mg weekly) | DAS28 ≤3.2 at week 39 and DAS28 <2.6 at week 52) | Dose decrease by 50% or discontinuation. Randomised 1:1 | DAS28 <2.6 | 37, 60 and 77% in low-dose ETN, MTX only and PBO groups (week 39) |  |
| Allaart et al. 2013 (BeSt)  (14) | Early RA | RCT | 508 | IFX (3 mg/kg) NB, four initial strategies existed in this trial that could all lead to IFX treatment | DAS ≤2.4 for ≥4 months | Discontinuation | DAS28 >2.4 | 48.1% (17 months) | 100% achieved DAS ≤2.4 |
| ***Established RA*** | | | | | | | | | |
| Huizinga et al. 2014 (ACT-RAY)  (36) | RA | RCT | 556 | TCZ (8 mg/kg/4 weeks) | DAS28-ESR <2.6 at two consecutive visits, 12 weeks apart | Discontinuation | Investigator’s discretion | 84% (52 weeks) | Moderate or high DAS28-ESR following restart at 4, 8 and 12 weeks, respectively: 29.7%, 18.6% and 11.7% |
| Kaine et al. 2012 (ALLOW)  (47) | RA | RCT | 167 | ABA (10 mg/kg loading dose then 125 mg weekly) | DAS28 reduction ≥0.6 from baseline at day 78 | Discontinuation | - | - | "Improvements seen within 1 month" Difference in DAS28 mean change of 0.1 at month 9 between placebo (-2.32) and ABA  (-2.22) |
| Haschka et al. 2015 (RETRO)  (35) | RA | RCT | 101 | ETN (50 mg weekly) ADA (40 mg eow) IFX (3 mg/kg) GOL (50 mg monthly) CTZ (200mg eow) TCZ (162 mg weekly) | DAS28-ESR <2.6 for ≥6 months in three sequential visits | Dose decrease by 50% Spacing by 50% Discontinuation (randomised 1:1:1) | DAS28-ESR >2.6 | 38.9% (12 months) tapering arm; 51.9% (12 months) discontinuation arm | - |
| van Herwaarden et al. 2014  ACR 500  (48) | RA | RCT | 118 | ADA (40 mg eow) ETN (50 mg weekly) | DAS28 <3.2 or clinical judgment of the rheumatologist | Increasing interval between injections every 3 months until flare or discontinuation | Dose reduction not possible | ADA - 50% (18 months) ETN - 36% (18 months) | - |
| van Herwaarden et al. 2014  ACR 1843  (33) | RA | RCT | 180 | ADA (40 mg eow) ETN (50 mg weekly) | DAS28< 3.2 or clinical judgment of the rheumatologist | Increasing interval between injections every 3 months until flare or discontinuation | DAS28-CRP increase >1.2 or DAS28-CRP increase >0.6 AND DAS28-CRP ≥3.2 | 10% (18 months) | - |
| Van Vollenhoven et al. 2013 EULAR FRI0815  (49) | RA | RCT | 73 | ETN (50 mg weekly) | DAS28 ≤3.2 for ≥11 months | Dose decrease by 50% | DAS28 ≥3.2 and an increase in DAS28 ≥0.6 or disease progression as determined by the investigator or patient | 56% (week 48) | - |
| Smolen et al. 2013 (PRESERVE) (50) | RA | RCT | 604 | ETN (50 mg weekly) | DAS28 ≤3.2 for 24 weeks | Dose decrease by 50% Discontinuation Randomised 1:1 | DAS28 ≤3.2 | 20.9% (week 88) tapering arm; 57.8% (week 88) discontinuation arm | - |
| van der Ven et al. 2014 ACR 120  (27) | RA | RCT | 67 | ADA (40 mg eow) ETN (50 mg weekly) | DAS44 <2.4 and SJ C≤1 | "Tapering" |  | - | - |
| Fautrel et al. 2016 (STRASS) (21) | RA | RCT | 138 | ETN (50 mg weekly) ADA (40 mg eow) | DAS28 ≤2.6 for >6 months and no structural damage progression observed on hand or foot X-rays in the year before inclusion | Step 0: current dosing Step 1: ETN 50 mg/10days or ADA 40 mg/21 days Step 2: ETN 50 mg eow or 40 mg/28 days Step 3: ETN 50 mg/21 days or ADA 40 mg/42 days Step 4: Discontinue | DAS28>2.6 with DAS28 increase >0.6 | 76.6% (28.1% at step 0; 10.9% at step 1; 10.9% at step 2; 21.9% at step 3; 1.6% at step 4) | 40.8% achieved remission (DAS28 ≤2.6); 38.8% LDA (not defined); 8.2% MDA (not defined) |
| Tanaka et al. 2015 (HONOR)  (24) | RA | Non-randomised trial | 197 | ADA (40 mg eow) | DAS28-ESR <2.6 for ≥6 months, without glucocorticoids, NSAIDs, coxibs and stable dose of MTX for ≥12 weeks | Discontinuation | Remission:DAS28-ESR <2.6 Flare: DAS28-ESR ≥ 3.2 | Remission: 52% (1 year); Flare: 40% (1 year) | 90% achieved LDA (DAS28-ESR <3.2) within 6 months and 100% within 9 months |
| Quinn et al. 2005  (51) | RA | Placebo-controlled pilot study | 20 | IFX (3 mg/kg - induction then every 8 weeks) | As per the trial design – no specific entry criteria for discontinuation | Discontinuation | Sustained ACR responses | 30% (12 months) | - |
| Chatzidionysiou et al. 2012 ACR 776  (52) | RA | Placebo-controlled pilot study | 33 | ADA (40 mg eow) | DAS28 <2.6 for ≥3 months | Discontinuation | Remission: DAS28 <2.6  Flare: DAS28 >2.6 or increase in DAS28 >1.2 from baseline at any point | Remission: 67% (week 28); Flare: 80% (28 weeks) | - |
| Raffeiner et al. 2015  (53) | RA | Prospective | 524 | ETN (25 mg bi-weekly) | DAS28 <2.6 for ≥12 months | Spacing by 50% (25 mg biweekly to 25 mg once weekly) | DAS28 <2.6 | 18.2% (3.6±1.5 years) | "Most of the patients failing dose reduction regained remission (DAS28 <2.6) with standard dose" 24.1% were switched to other biologic agents |
| Takeuchi et al. 2014 (39) | RA | Prospective | 51 | ABA (10 mg/kg or 2 mg/kg) | DAS28-CRP <2.3 | Discontinuation | DAS28-CRP <2.3 | 58.8% (week 52) | DAS28-CRP decreased by 1.3 within 12 and 24 weeks |
| Nawata et al. 2008  (54) | RA | Prospective | 172 | IFX (3 mg/kg - induction then 8 weekly) | DAS28-ESR <2.6 for >24 weeks | Discontinuation | DAS28-ESR <2.6 | 82.7% (24 weeks) | - |
| Iwamoto et al. 2014  (55) | RA | Prospective | 42 | ETN (50 mg weekly) ADA (40 mg eow) IFX (3 mg/kg) GOL (50 mg monthly) CTZ (200mg eow) TCZ (8 mg/kg/4 weeks) | DAS28 <2.6 | Discontinuation | DAS28 >3.2 | 40% (1 year) | - |
| Naredo et al. 2014  EULAR THU0250  (56) | RA | Prospective | 77 | ETN (50 mg weekly) ADA (40 mg eow) IFX (3 mg/kg) GOL (50 mg monthly) ABA (10 mg/kg) TCZ (8 mg/kg/4 weeks) | Stable dose of biologic for >12 months; sustained clinical remission judged by rheumatologist and DAS28/SDAI for >12 months; ≤5 mg/day of prednisone for >6 months; no NSAID for >1 week; no local corticosteroid injections for >6 months | "Agreed strategy with physician" | An increase in BT doses by their usual consultant rheumatologist and/or the presence of clinical disease activity according to both DAS28 and SDAI criteria | 29.9 and 45.5% (6 and 12 months) | - |
| Tanaka et al. 2010 (RRR)  (37) | RA | Observational | 114 | IFX (3 mg/kg/8 weeks) | DAS28-ESR <3.2 for >24 weeks, <5mg/day prednisolone | Discontinuation | DAS28-ESR <3.2 | 45% (1 year) | Estimated 90.1% achieved a DAS28 <4 |
| Kamiya et al. 2013  EULAR SAT0124  (57) | RA | Observational | 80 | ETN (50 mg weekly initially tapered to 25 mg eow) | DAS28-ESR <2.6 for >6 months | Discontinuation |  | - | - |
| van der Maas  et al. 2012  (58) | RA | Observational | 51 | IFX (3 mg/kg) | DAS28 <3.2 and stable treatment for ≥6 months | 25% dose decrease every 8–12 weeks - if no flare then discontinuation | Down titrate | 39% | - |
| Alivernini et al. 2014 EULAR FRI0276  (59) | RA | Observational | 49 | ADA (40 mg eow) ETN (50 mg weekly) | DAS <1.6 | Spacing by 50% If PD- after 3 months spacing, discontinuation | Increase of DAS28 > 1.2 from DAS value at last ultrasound assessment | 30.9% (3 months after tapering); 20% (9 months after discontinuation) | - |
| Nishimoto et al. 2014 (DREAM)  (60) | RA | Observational | 187 | TCZ (8 mg/kg/4 weeks or 4/mg/kg/4weeks or 2 mg/kg/4 weeks) | DAS28-ESR ≤3.2 at two or three consecutive visits | Discontinuation | DAS28-ESR <2.6 | 85.8% (52 weeks) | - |
| Brocq et al. 2009  (40) | RA | Observational | 304 | IFX (3 mg/kg) ETN (50 mg weekly) ADA (40 mg eow) | DAS28 <2.6 for >6 months (confirmed by 2 visits 6 months apart), without NSAIDs, prednisolone ≤5 mg/day | Discontinuation | DAS28 >3.2 | 40, 55, 70 and 75% (3, 6, 9 and 12 months) | 100% achieved remission (DAS28 <2.6); 86.7% at 2 months |
| Plasencia et al. 2014  EULAR THU0157  (61) | RA | Observational | 144 | ETN (50 mg weekly) ADA (40 mg eow) IFX (3 mg/kg) | DAS28 <3.2 for >6 months | "Dose reduction and/or interval elongation - at visit 2 spacing increase of 32.8% IFX, 52.9% ADA and 52.6% ETN" | Not defined | 41.8% | - |
| Marks et al. 2015  (28) | RA | Observational | 70 | ETN (50 mg weekly) ADA (40 mg eow) IFX (3 mg/kg) GOL (50 mg monthly) CTZ (200 mg eow) | DAS28 <2.6 for >6 months, no corticosteroids and no synovitis on US | Spacing by 33% Dose decrease by 33% | Flare: Not defined  Remission: DAS (DAS28 <2.6) and US remission (PDUS < 1) | Flare: 46% (7.65 months); Remission: 4, 37, 63 and 67% (3, 6, 9 and 18 months) | 19% DAS (DAS28 <2.6) and US remission (PDUS <1); 19% DAS remission; 47% LDA (DAS28 <3.2 and PDUS ≤1) |
| Mamoto et al. 2013 ACR1449  (62) | RA | Observational | 26 | ETN (50 mg weekly and 25 mg weekly) | Unclear | Dose decrease by 50% | PDUS score significantly higher in 25 mg weekly group (P<0.05) | - | - |
| Chatzidionysiou et al. 2013 ACR 500 (34) | RA | Registry | 800 | RTX (dose unclear) | Unclear | Discontinuation |  | - | - |
| Greenberg JD et al. 2014  EULAR THU0174  (63) | RA | Registry | 685 | ETN (50 mg weekly or 25 mg biweekly) | "LDA or remission" | Reduced dose defined as "<50 mg/week or equivalent" | LDA: not defined  Remission: not defined | LDA: OR 0.62 and 0.16 (6 and 12 months) for reduced vs standard dose;  Remisison: OR 1.21 and 0.64 (6 and 12 months) for reduced vs standard dose | - |
| Kavanaugh et al. 2013 ACR 1425  (38) | RA | Registry | 717 | TNF inhibitors | CDAI ≤10 | Discontinuation | CDAI ≤10 | 2.3, 26.6, 44.4, 57.8, 72.4% (6, 12, 18, 24 and 36 months) | - |
| van Herwaarden et al. 2014  (64) | RA | Retrospective | 22 | TCZ (8 mg/kg) | DAS28 <3.2 and/or physician judgement | Decrease in dose by 50% | DAS28 <3.2 and/or physician judgement | 33 and 45% (3 and 6 months) | 88.9% achieved LDA (DAS28 <3.2) |
| Harigai et al. 2012  (65) | RA | Retrospective | 46 | ADA (40 mg eow) | DAS28-CRP <2.7 | Discontinuation |  | 36.4% had to restart biologics by week 52; 18.2% not LDA (DAS28-CRP <2.7) at week 52 | - |
| ***Axial SpA*** | | | | | | | | | |
| Arends et al. 2015  (43) | AS | Prospective | 58 | ETN (25 mg biweekly or 50 mg weekly) ADA (40 mg eow) IFX (5 mg/kg/6 weeks or 5 mg/kg/8weeks) | BASDAI <4 for >6 months | ETN: if 25 mg biweekly starting, stepwise to 25 mg/5 days then 25 mg weekly then 25 mg/10days then 25 mg eow; or if 50 mg weekly starting, stepwise to 50 mg/10 days then 50 mg eow ADA: 40 mg/21 days then 40 mg/28 days IFX: if 5 mg/kg/6 weeks starting, stepwise to 3 mg/kg/6 weeks; or if 5 mg/kg/8 weeks starting, stepwise to 3 mg/kg/8 weeks then 3 mg/kg/10 weeks | Not remaining on dose reduction | 26, 38, 43 and 47% (6, 12, 18 and 24 months) | 88% reached BASDAI <4 6–12 months after returning to dose |
| De Stefano et al. 2014  (41) | AS | Prospective | 38 | ETN (25 mg biweekly) | Value of <2 on each of four ASAS domain; score BASDAI <2, absence of peripheral arthritis e/o enthesitis; absence of inflammatory extra-articular manifestations; and normalization of CRP, without taking any additional  NSAlDs and corticosteroids | Dose decrease by 50% | Value of <2 on a 1-10 point scale in each of four ASAS domain; score BASDAI <2, absence of peripheral arthritis e/o enthesitis; absence of inflammatory extra-articular manifestations; and normalisation of CRP, without taking any additional drug including NSAlDs and corticosteroids | 53% (week 46) | - |
| Závada et al. 2014  (44) | AS | Prospective | 386 | ETN (50 mg weekly) ADA (40 mg eow) IFX (3 mg/kg) | BASDAI <4 following >6 months of treatment | Median of 50% reduction either by dose decrease or spacing | Various BASDAI cut-offs | 11.3% (12 months) | - |
| Almirall et al. 2013 EULAR THU0368  (42) | Axial SpA | Observational | 40 | ETN (50 mg weekly) ADA (40 mg eow) IFX (3 mg/kg) | BASDAI ≤2, absence of arthritis and enthesitis, CRP normal for >6 months without NSAIDs | ETN: decrease in dose by 50% or spacing by 50% IFX every 8 weeks ADA spacing by 50% | Remission: BASDAI ≤2 Relapse: BASDAI ≥4 | Remission: 15 and 29% ( 3 and 6 months); Relapse: 5 and 16.5% (3 and 6 months) | - |

| ***PsA*** | | | | | | | | | |
| --- | --- | --- | --- | --- | --- | --- | --- | --- | --- |
| Huynh et al. 2014 ACR 1594  (45) | PsA | Registry | 325 | TNF inhibitors | CDAI ≤10 and skin psoriasis PGA ≤20/100 | Discontinuation | CDAI >10 or increase in skin assessment >20 or increase on concomitant DMARD or prednisone dose or start DMARD, prednisone or biologic | 44.9% (29.2 months) | - |
| ***Cross indication*** | | | | | | | | | |
| Inciarte-Mundo et al. 2014  (66) | RA AS PsA JIA | Retrospective | 153 | ETN (50 mg weekly) ADA (40 mg eow) IFX (3 mg/kg) TCZ (8 mg/kg) | As per clinician's choice | IFX 5 mg/kg/9 or 10 weeks ETN 50 mg/10 or 15 or 21 days and 25 mg/7 days ADA @ 40 mg/21 or 30 days TCZ @ 6 or 4 mg/kg/4 weeks. |  | - | - |

Where exact definitions have not been provided, a quote from the relevant study has been added to the table contained within quotation marks to demonstrate it is an exact replication of what is in the study. ABA, abatacept; ACR*,* American College of Rheumatology; ADA, adalimumab*;* ASAS, Assessment of SpondyloArthritis international Society; AS, ankylosing spondylitis; axial SpA, axial spondyloarthritis*;* PBO*,* placebo; PsA*,* psoriatic arthritis*;* BASDAI, Bath Ankylosing Spondylitis Disease Activity Index; CTZ, certolizumab pegol; CDAI, Clinical Disease Activity Index; CRP, C-reactive protein; DAS, disease activity score*;* DMARD, disease-modifying anti-rheumatic drugs; EOW, every other week; ESR, erythrocyte sedimentation rate; ETN, etanercept; RTX, rituximab; GOL, golimumab; JIA*,* juvenile idiopathic arthritis*;* LDA, low disease activity; MDA, minimal disease activity*;* MTX, methotrexate; NSAIDs, non-steroidal anti-inflammatory drugs; OR, odds ratio; PDUS, power doppler ultrasonography; RA, rheumatoid arthritis; RCT, randomised controlled trial; SJC, swollen joint count; TCZ, tocilizumab; TNF-α, tumour necrosis factor alpha; US, ultrasound.
